# The Traditional Uses, Phytochemistry, Pharmacokinetics, Pharmacology, Toxicity, and Applications of *Corydalis saxicola* Bunting: A Review

**DOI:** 10.3389/fphar.2022.822792

**Published:** 2022-02-16

**Authors:** Yanru Guo, Linjun Zhao, Botao Chang, Jia Yu, Jiangping Bao, Qi Yao, Jun Luo

**Affiliations:** ^1^ Department of Pharmacy, The First Affiliated Hospital of Guizhou University of Traditional Chinese Medicine, Guiyang, China; ^2^ College of Graduate, Guizhou University of Traditional Chinese Medicine, Guiyang, China; ^3^ Xintian Community Health Service Center of Guiyang, Guiyang, China; ^4^ Department of Anesthesiology, The First Affiliated Hospital of Guizhou University of Traditional Chinese Medicine, Guiyang, China

**Keywords:** Corydalis saxicola bunting, phytochemistry, pharmacokinetics, pharmacology, toxicity, traditional uses

## Abstract

**Background:** *Corydalis saxicola* Bunting (CSB) is a perennial herb belonging to genus *Corydalis* (Papaveraceae), called “Yan-huang-lian” in the Chinese folk. Traditionally, it is used to treat acute conjunctivitis, corneal pannus, acute abdominal pain, hemorrhoidal bleeding, haematochezia, swelling, hepatitis, cirrhosis and liver cancer based on traditional Chinese medicine (TCM) concepts.

**Purpose:** This review aims to summarize and analyze the pharmacokinetics, pharmacological and toxicological properties of CSB and its extracts; to highlight the relevance of modern pharmacology to traditional pharmacology; also to assess its therapeutic potential.

**Methods:** CSB related literatures were searched and screened from databases including PubMed, Web of Science and CNKI. The selected literatures provided reliable source identification evidences.

**Results:** In traditional medicine concepts, CSB has the effects of clearing away heat and detoxification, eliminating dampness, relieving pain, and stopping bleeding. Its modern pharmacology includes hepatoprotective, anticancer, anti-inflammatory, analgesic, antibacterial, anti-oxidative effects. Further, some pharmacological effects support its traditional uses. The CSB total alkaloids (CSBTA) are the main constituents isolated from this plant, and they exert the major of the pharmacological effects. Toxicological studies have shown that the toxicity of CSBTA is mild and reversible in rodents and beagle dogs.

**Conclusion:** Although the present study summarizes the botany, phytochemistry, pharmacokinetics, pharmacology, toxicity, and applications of this plant, it is still necessary to systemically evaluate the chemistry, safety and parameters related to drug metabolism of the extracts or compounds from this plant before or in clinical trials in the future. Meanwhile, cancers and inflammatory-related diseases may be new research directions of this ethnomedicine.

## Introduction

*Corydalis saxicola* Bunting (CSB) is a light green and soft perennial herb of *Corydalis* (family Papaveraceae). It grows in rock cliffs or alpine caves, and is mainly distributed in the south of China, including Guizhou, Guangxi, Yunnan and Sichuan provinces (Li et al., 2018a). Traditionally, the whole plant of CSB, Yan-huang-lian is used to cure diseases in the Chinese folk. It was firstly documented to have properties of clearing away heat and detoxication, removing dampness, relieving pain and hemostasis in a book named Guizhou Herbal Medicine. Nowadays, pharmacological studies have provided evidence that this ethnomedicine can treat liver diseases and also show that it also has important pharmacological activities such as anticancer, anti-inflammation (Liang et al., 2016; Xie et al., 2021). Meanwhile, CSBTA are the main active constituents to exert the pharmacological effects described as before.

Although this plant has been widely used, it has not been included in Chinese Pharmacopoeia until now. In addition, a systematic quality standard is still absent. Furthermore, the chemical, pharmacokinetic, pharmacological, toxicological studies on this plant need enhancement. Thus, CSB related literatures were searched in databases including PubMed, Web of Science and CNKI. Then the main keywords included “*Coryalis saxicola* Bunting,” or “*Corydalis saxicola* Bunting total alkaloids,” or “dehydrocavidine.” Subsequently, the botany, chemistry, pharmacology, toxicology, and applications of this plant were summarized and analyzed systemically in this study. In view of this, we expect to provide some cues for utilizing this plant more deeply in the future.

## Botanical Description

There are about 400 species of genus *Corydalis* (Papaveraceae) worldwide, mainly distributed in the northern temperate zone. Over 50% of the *Corydalis* are distributed in China, mostly in the southwest region (Li et al., 2018a). CSB grows in rock cliffs or alpine caves at a proper temperature of 0–30°C. Due to the harsh growth environment, wild CSB resources are insufficient. Several studies have given recommendations for the cultivation of this plant. However, in a recent study, wild and cultivated CSBs varied in composition (Xie et al., 2021).

**FIGURE 1:**
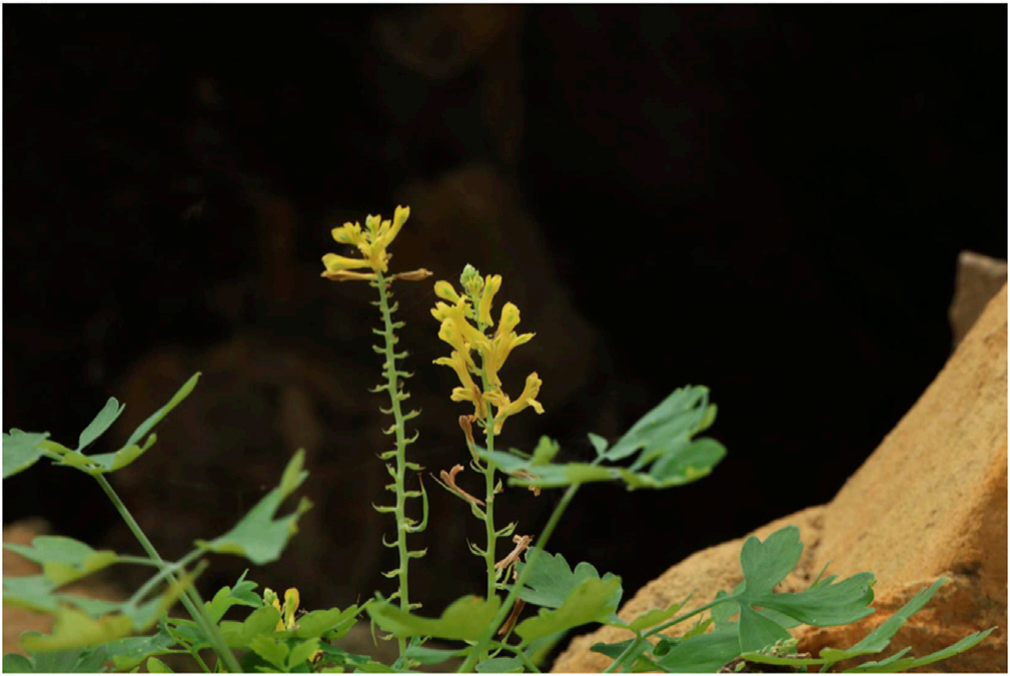
Plant morphology of CSB (Photographed and provided by Jun Luo).

This plant is 15–40 cm tall with well-developed taproots; stems are 1–3 cm; whole plant is glabrous and soft; they are tufted; petioles are long; leaves blade are triangular-ovate, pinnately compound, and deeply lobed, with pointed apex and coarse-toothed margin. Flowers are yellow, racemes, terminal or opposite leaves, 7–14 cm long. Floral bracts are elliptic-lanceolate, sepals 2, petals 4, stamens 6, stigma 2-lobed. Capsules are terete, slightly curved, and had 15–22 seeds (Figure 1). Most of seeds are round, and have cup-shaped caruncle covering half of the seed.

## Phytochemistry

Alkaloids were the main chemical constituents isolated from CSB. Among the 57 reported constituents, alkaloids accounted for 49 **(1–49)**. The remained eight were steroidal compounds **(50–57)** (Tang et al., 2018). The alkaloids constituents included 13 berberines **(1–13)**, 14 protoberberines **(14–27)**, six benzophenanthridines **(28–33)**, two protopines **(34–35)**, two benzyltetrahydroisoquinolines **(36–37)**, three aporphines **(38–40)**, two morphinines **(41–42)**, two simple indoles **(43–44)**, three organic amines **(45–47)**, one simple isoquinoline **(48)**, and one guanidinium salt **(49)**, among which isoquinoline alkaloids accounted for the largest proportion (Table 1).

**TABLE 1:** The compounds isolated from CSB.

No.	Name	Parts of plant	Source	Chemical structure	Reference
1	dehydrocavidine	Whole plant	Methanol extract	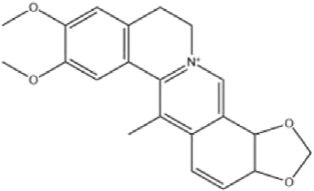	Li et al. (2008)
2	berberine	Whole plant	Ethanol extract	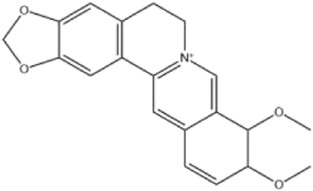	Cheng et al. (2008a)
3	palmatine	Roots	Ethanol extract	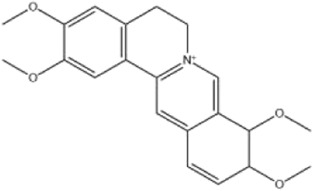	Wu et al. (2007)
4	dehydrocheilanthifoline	Whole plant	Ethanol extract	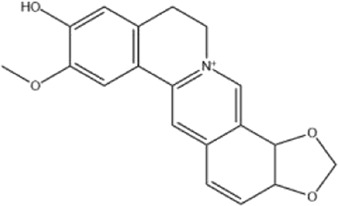	Cheng et al. (2008a)
5	coptisine	Roots	Ethanol extract	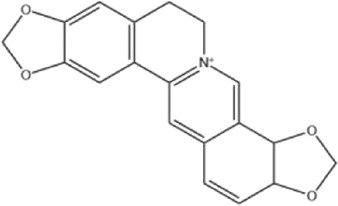	Wu et al. (2007)
6	jatrorrhizine	Whole plant	CSBTA extract	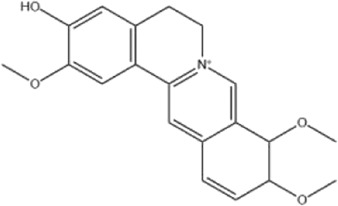	Wu et al. (2015)
7	columbamine	Whole plant	CSB extract	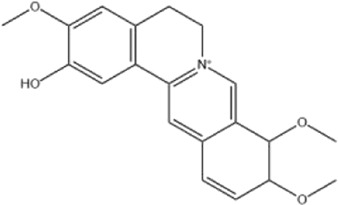	Zhou et al. (1989)
8	tetradehydroscoulerine	Whole plant	Ethanol extract	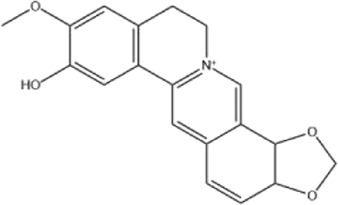	Li et al. (2006)
9	dehydrodiscretamine	Whole plant	Methanol extract	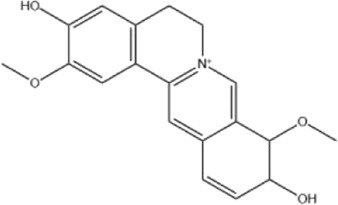	Cheng et al. (2008a)
10	dehydroisoapocavidine	Whole plant	Methanol extract	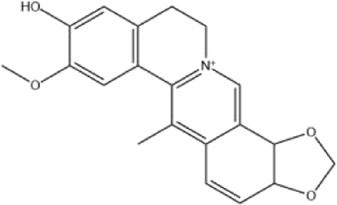	Li et al. (2008)
11	dehydroapocavidine	Whole plant	Methanol extract	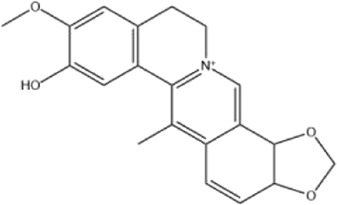	Cheng et al. (2008a)
12	corysamine	Whole plant	CSB extract	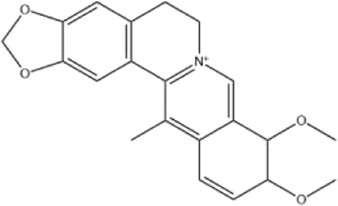	Zhou et al. (1989)
13	epiberberine	Whole plant	Methanol extract	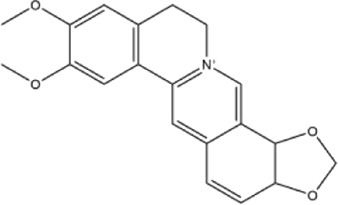	Xia, (2002)
14	cavidine	Whole plant	Methanol extract	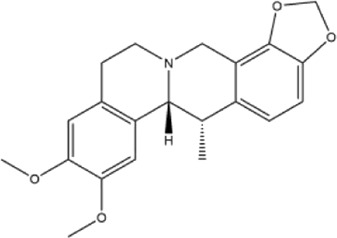	Huang et al. (2012)
15	corydaline	Roots	Ethanol extract	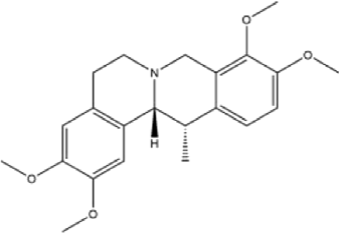	Wu et al. (2007)
16	stylopine	Roots	Ethanol extract	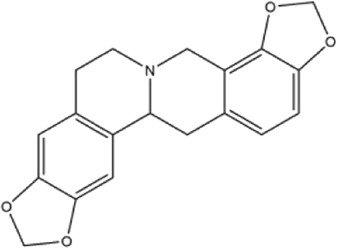	Wu et al. (2007)
17	cheilanthifoline	Whole plant	Ethanol extract	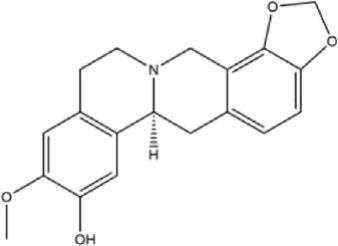	Cheng et al. (2008a)
18	tetrahydrocolumbamine	Whole plant	Ethanol extract	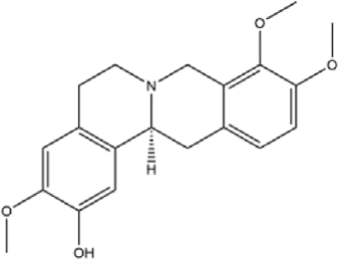	Ke et al. (1982)
19	tetrahydropalmatine	Roots	Ethanol extract	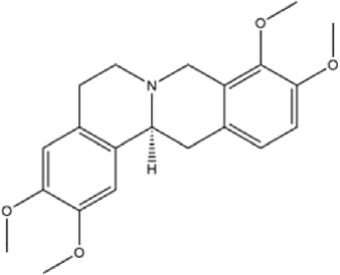	Wu et al. (2007)
20	canadine	Whole plant	Ethanol extract	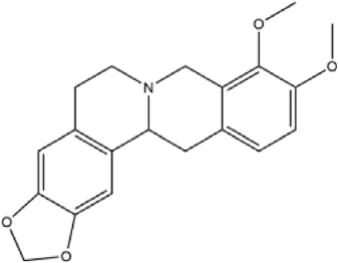	He et al. (2014)
21	scoulerine	Whole plant	Ethanol extract	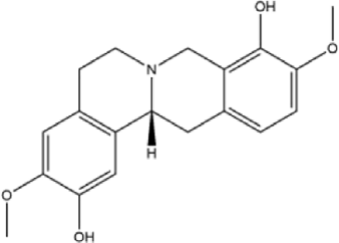	Cheng et al. (2008a)
22	β-hydroxystilopine	Whole plant	Ethanol extract	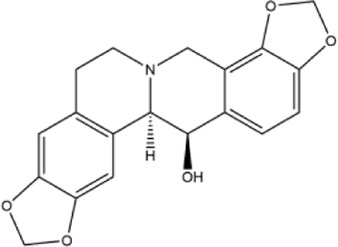	Ke et al. (1982)
23	(+)-thalictrifoline	Whole plant	Methanol extract	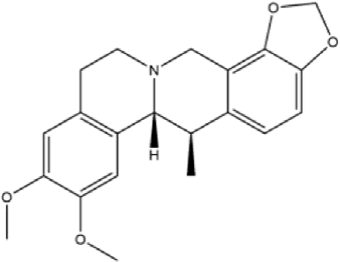	Huang et al. (2012)
24	(−)-corynoxidine	Whole plant	Methanol extract	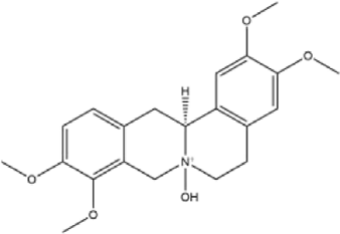	Huang et al. (2012)
25	4-nitroisoapocavidine	Whole plant	Methanol extract	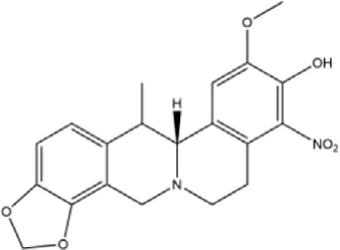	Huang et al. (2012)
26	(+)-1-nitroapocavidine	Whole plant	Methanol extract	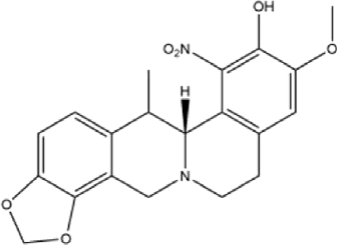	Huang et al. (2012)
27	2,9-dihydroxy-3,11-dimethoxy-1,10-dinitrotetrahydroprotoberberine	Whole plant	Methanol extract	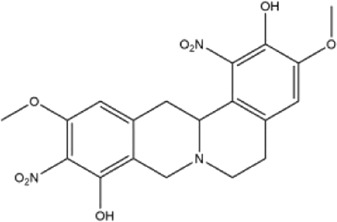	Huang et al. (2012)
28	sanguinarine	Whole plant	Methanol extract	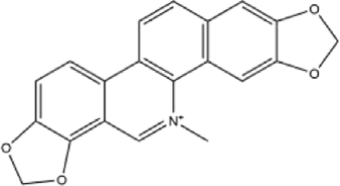	Huang et al. (2012)
29	dihydrosanguinarine	Roots	Ethanol extract	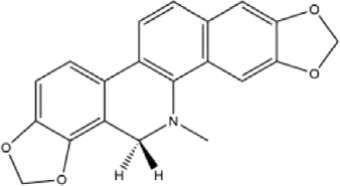	Wu et al. (2007)
30	dihydrochelerythrine	Roots	Ethanol extract	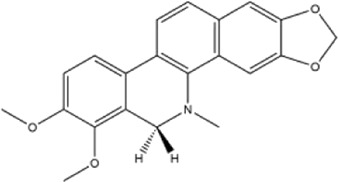	Wu et al. (2007)
31	chelerythrine	Whole plant	Ethanol extract	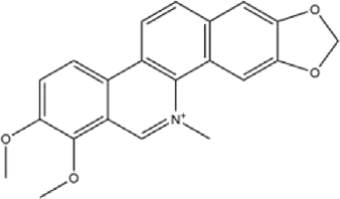	Cheng et al. (2008a)
32	6-acetonyl-5,6-dihydrosanguinarine	Roots	Ethanol extract	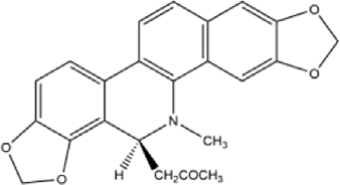	Wu et al. (2007)
33	8-acetonyldihydrochelery-thrine	Whole plant	Methanol extract	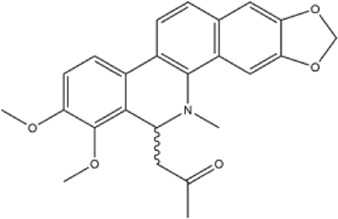	Huang et al. (2012)
34	protopine	Whole plant	Ethanol extract	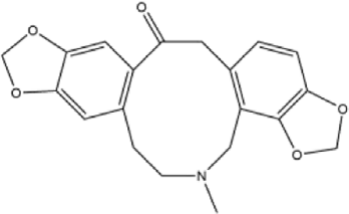	He et al. (2014)
35	allocryptopine	Whole plant	Ethanol extract	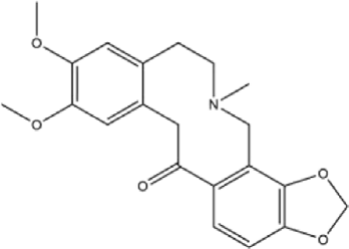	Wang et al. (2007)
36	adlumidine	Roots	Ethanol extract	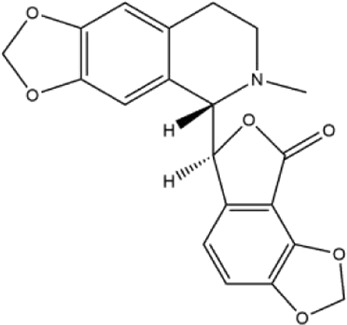	Wu et al. (2007)
37	oxyacanthine	Whole plant	Ethanol extract	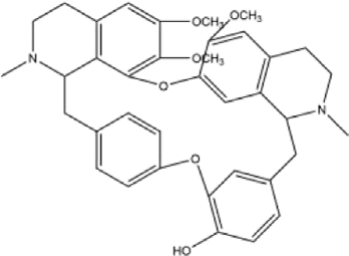	Wang et al. (2007)
38	(+)-isocorydine	Whole plant	Methanol extract	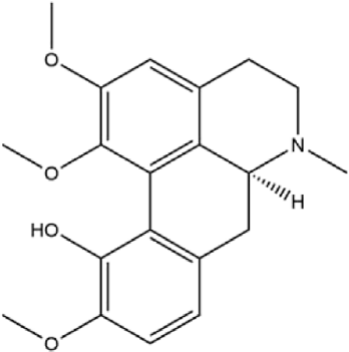	Cheng et al. (2008a)
39	(+)-magnoflorine	Roots	Ethanol extract	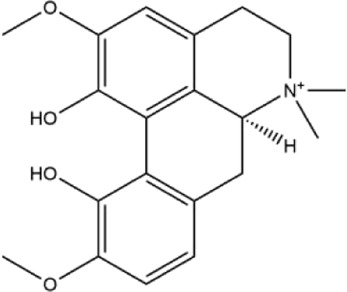	Wu et al. (2007)
40	saxicolaline A	Roots	Ethanol extract	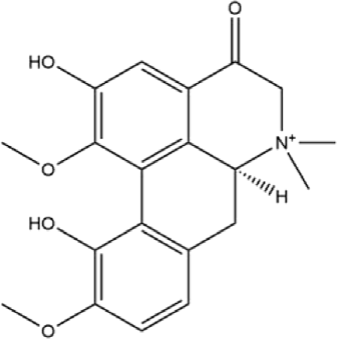	Wu et al. (2007)
41	pallidine	Whole plant	Ethanol extract	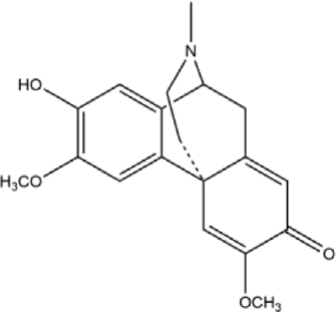	He et al. (2014)
42	(−)-salutaridine	Roots	Ethanol extract	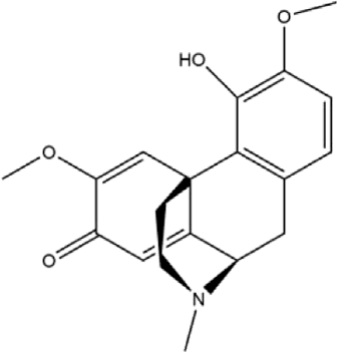	Wu et al. (2007)
43	2,3-dihydro-5-methoxy-6-methyl-1H-indole	Whole plant	Methanol extract	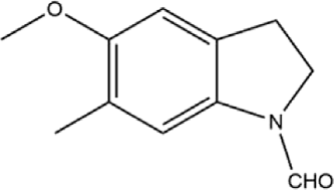	Li et al. (2008)
44	2,3-dihydro-2-hydroxy-5-methoxy-6-methyl-1H-indole	Whole plant	Methanol extract	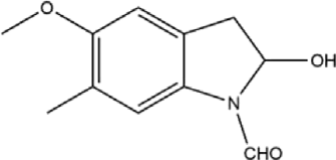	Li et al. (2008)
45	14-amino-27ane	Whole plant	Ethanol extract	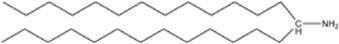	Mao, (2006)
46	14-amino-28ane	Whole plant	Ethanol extract	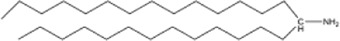	Mao, (2006)
47	N-Methylnarceimicine	Roots	Ethanol extract	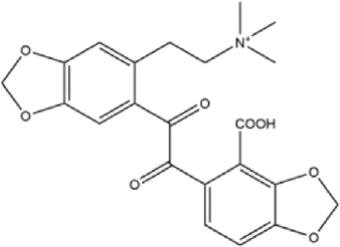	Wu et al. (2007)
48	corypalline	Whole plant	Ethanol extract	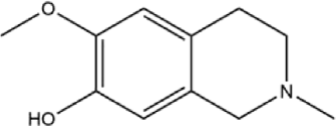	Cheng et al. (2008a)
49	feruloylagmatine	Whole plant	Ethanol extract	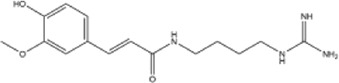	Cheng et al. (2008a)
50	cholesterol	Whole plant	Ethanol extract	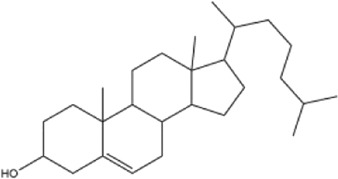	Mao, (2006)
51	β-sitosterol	Whole plant	Ethanol extract	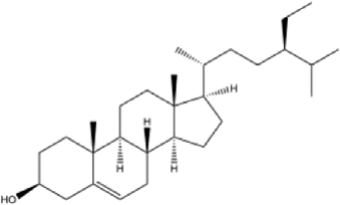	Wang et al. (2007)
52	cycloeucalenol	Whole plant	Ethanol extract	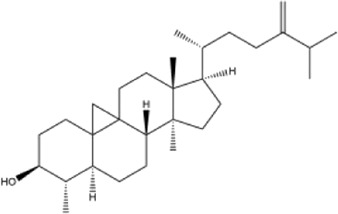	Wang et al. (2007)
53	betulinic acid	Whole plant	Ethanol extract	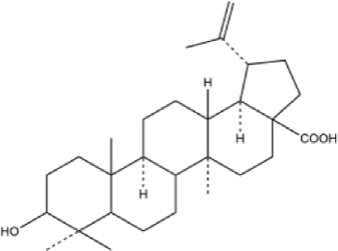	Wang et al. (2007)
54	oleanolic acid	Whole plant	Ethanol extract	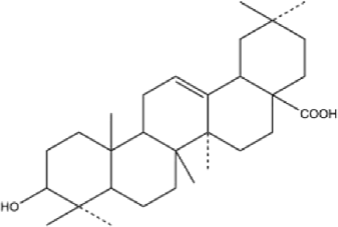	Wang et al. (2007)
55	betuline	Whole plant	Ethanol extract	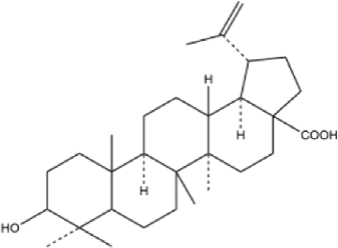	Wang et al. (2007)
56	β-amyrin acetate	Whole plant	Ethanol extract	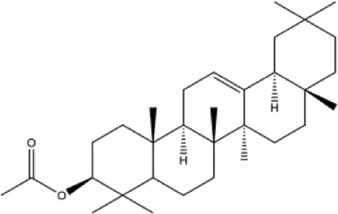	Wang et al. (2007)
57	daucosterol	Whole plant	Ethanol extract	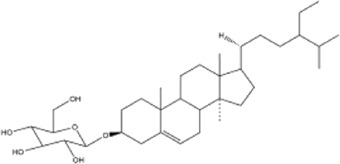	Wang et al. (2007)

Alkaloids are a class of nitrogen-containing basic organic compounds in nature. They possess the properties of alkali-like and cyclic structures mostly. Alkaloids show a variety of pharmacological activities, including anti-oxidative, anticancer, lipid-lowering, hypoglycemic, antibacterial, and other effects (Cicero and Ertek, 2009; Cicero and Baggioni, 2016; Kukula-Koch and Widelski, 2017). Its nitrogen atom and cyclic structure are responsible for the main pharmacological activities (Xu et al., 2020). As we know, isoquinoline alkaloids are one of the relatively abundant alkaloids (Singh et al., 2021). Currently, most of the alkaloids isolated from CSB are isoquinolines, among which dehydrocavidine is a characteristic and one of the most abundant constituents in this plant.

Cheng et al. evaluated the quality of extracted compounds from CSB using a HPLC-DAD method (a Gemini™ C_18_ column, 5 μm, 250 × 4.6 mm i.d., Phenomenex Inc., CA, USA) with a gradient solvent system (20 mM aqueous ammonium acetate–acetonitrile with a flow-rate of 1.0 mL/min) at 270 and 280 nm (Cheng et al., 2008b). The dehydrocavidine content isolated from 12 batches of CSB samples from different habitats accounted for 32.71–62.83% (8.84–19.77 mg/g) of the total alkaloids. Tang et al. established the HPLC fingerprint of CSB using the Agilent Eclipse XDB-C18 (4.6 mm × 250 mm, 5 μm) column (acetonitrile-0.1% formic acid as mobile phases, gradient elution at a flow rate of 0.5 mL/min) (Tang et al., 2019). The results showed that the similarity of 10 batches of CSB samples from different habitats was above 90%. The dehydrocavidine content in the quantitative analysis ranged from 7.85 to 12.71 mg/g. Generally, the content of characteristic constituent dehydrocavidine was determined to evaluate the quality of the plant (Deng et al., 2009). However, it is still urgent to establish a complete set of quality standards for this plant.

## Pharmacokinetics

Pharmacokinetics reflects the metabolic process of drugs *in vivo*, which is affected by different administration methods. Traditional Chinese medicine is mostly administered orally. Therefore, we prefer to identify the evidence supporting its oral effectiveness. Existing studies on the pharmacokinetics of CSB have focused on the monomer constituents. It is known that they are metabolized mainly through the liver and intestines.

Li et al. developed an HPLC coupled with tandem mass spectrometry method for simultaneously quantitating dehydrocavidine, coptisine, dehydroapocavidine and tetradehydroscoulerine in plasma and urine (Li et al., 2006). The results showed the different pharmacokinetic parameters of these four alkaloids in the two states of intravenous and oral administration (Table 2). For intravenous administration, the systemic clearance of these four alkaloids was 180, 147, 111 and 93% of hepatic blood flow in rats, respectively. They could be quickly cleared by the liver *in vivo*. The major alkaloid constituents of CSB are metabolized by hepatic cytochrome P450s (Yu et al., 2018). Subsequent study by Dai also showed that the liver in pathological states affects the pharmacokinetic parameters of the alkaloid constituents of CSB (Dai et al., 2018). In addition, they had high volumes of distribution (>15.6 L/kg). In all, these alkaloids are metabolized by the liver and are widely distributed in the body. For oral administration, the maximum blood concentrations of the four alkaloids were 88.4 ± 29.8, 19.0 ± 6.52, 115 ± 52.2, and 13.8 ± 5.72 ng/mL. The data were consistent with the proportion of each constituent in the total extract. Correspondingly, their oral bioavailabilities were 13.24 ± 10.86%, 7.21 ± 5.06%, 9.88 ± 6.3% and 10.47 ± 5.42%, respectively. Despite the first pass effect caused by liver, they are still absorbed after the oral administration, which suggests the oral use of CSB. However, the specific metabolic pathway of CSB is yet to be clarified. Other monomeric constituents with pharmacological effects also need more metabolic evidences to support their oral availabilities.

**TABLE 2:** The pharmacokinetic parameters of the four components isolated from CSB in rats.

Parameters	dehydrocavidine	coptisine	dehydroapocavidine	tetradehydroscoulerine
**Usage 1**	**Intravenous injection, 10 mg/kg**			
t_1/2β_ (min)	207 ± 27.6	288 ± 112	214 ± 104	253 ± 170
CL (L/min/kg)	0.10 ± 0.02	0.08 ± 0.03	0.06 ± 0.01	0.05 ± 0.01
Vd (L/kg)	27.8 ± 3.78	30.1 ± 9.38	16.9 ± 5.99	15.6 ± 6.81
AUC_0–480_ (mg/L min)	38.5 ± 8.38	14.6 ± 3.49	59.4 ± 11.8	8.68 ± 1.60
AUC_0–∞_ (mg/L min)	42.1 ± 9.41	20.4 ± 6.54	68.0 ± 16.0	10.1 ± 1.93
**Usage 2**	**Oral administration, 10 mg/kg**			
t_1/2β_ (min)	154 ± 94.51	309 ± 157.2	146 ± 101.88	312 ± 278.71
AUC_0–240_ (mg/L min)	4.61 ± 3.21	1.47 ± 1.03	6.72 ± 4.29	1.06 ± 0.549
AUC_0–∞_ (mg/L min)	5.57 ± 4.57	1.47 ± 1.03	6.72 ± 4.29	1.06 ± 0.549
F (%)	13.2 ± 10.9	7.21 ± 5.06	9.88 ± 6.3	10.5 ± 5.42
C_max_ (ng/mL)	88.4 ± 29.8	19.0 ± 6.52	115 ± 52.2	13.8 ± 5.72
T_max_ (min)	15.0 ± 0	13.8 ± 2.5	13.8 ± 2.5	13.8 ± 2.5

Liu et al. determined dehydrocavidine to explore the characteristics and mechanism of CSBTA absorption in the gastrointestinal tracts of rats by using systemic intestinal circulation and unidirectional perfusion (Liu et al., 2009). They found that CSBTA were absorbed in the digestive tract. At middle (0.20 g/L) and high (1.00 g/L) concentrations, the absorption mechanism was passive diffusion independent of the concentration, which was consistent with the study of Shi (Shi et al., 2013). It also showed that an efflux mechanism of transporters during intestinal mucosal transport of dehydrocavidine. A recent study has confirmed that CSB undergoes the intestinal metabolism (Wu et al., 2021). After intragastric administration of CSB in rats, metabolites of dehydrocavidine, palmatine and berberine were found in blood, urine, bile and feces. These three alkaloids underwent methylation, hydroxylation, demethylation, reduction, glucuronidation and sulfation reactions *in vivo*. In conclusion, the characteristic metabolism of CSB extract *via* the liver and intestine after oral administration reasonably explains the use of this ethnomedicine in liver-related disease. In future, oral formulation development may be a favorable direction for the application.

## Pharmacological Effects

The pharmacological effects of constituents or extracts from CSB were investigated and displayed in Table 3.

**TABLE 3:** The pharmacological effects of CSB.

Pharmacological effects	Component	Detail	Cell lines/model	Administration	Effective dosage	Reference(s)
Hepatoprotective effects	CSBTA	Reduce liver ALT, AST, Hyp, MDA, TGF-β1 and MMP-9 levels; restore TP, ALB and SOD levels	Wistar rats	i.g.	75 and 100 mg/kg	Liang et al. (2008)
		Improve liver hypertrophy and fatty lesions; reduce serum TC, TG, LDL-C and NEFA levels; regulate AMPK/PI3K/Akt pathway	Male C57BL/6 mice	i.g.	25 and 100 mg/kg	Chen et al. (2021)
	Dehydrocavidine	Reduce serum ALT, AST, ALP, TBIL and Hyp levels; restore GPx, CAT and SOD levels; regulate Bcl2, Cyp3a13, IL18 and Rad50 genes	Sprague Dawley rats	i.p.	0.5 and 1.0 mg/kg	Wang et al., 2008; Wang et al., 2011
		Reduce liver TGF-β1, Bcl2 expression; increase Bax and Caspase-3 expression	HSC-T6 cells	—	0.01–0.10 mg/mL	Lu et al. (2017)
	CSB extract	Interferes with amino acid, glucose, lipid metabolism and other metabolic pathways	Male Sprague Dawley rats	i.g.	2.5 g/kg	Liang, et al., 2016; Tang and Guangxi, 2017
		Increase liver BESP, NTCP expression; improve the enterohepatic circulation of bile acid	Male Wistar rats	i.g.	10 mg/kg	Liu et al. (2019b)
		Reduce serum DHBV-DNA, ALT and AST levels	Guangxi brown spot ducklings	i.p.	8 mg/kg	Wang et al. (2009)
	Dehydrocheilanthifoline	Inhibite the secretion of HBsAg and HBeAg; reduce HBV DNA levels	HepG2 cells	—	3.13, 6.25, 12.50 and 25.00 μM	Zeng et al. (2013)
Antitumor effects	CSBTA	Inhibit cellular telomerase activity	Tca8113 cells	—	0.050 and 0.100 g/L	Li et al. (2007)
		Inhibit NF-κB P50 and P65 subunits expression	Tca8113 cells	—	0.1 and 0.2 g/L	Xu and Liao, (2010)
		Inhibit Bcl2 expression	Tca8113 cells	—	0.200 and 0.300 g/L	Zhu and Liao, (2011)
		Increase E-cadherin expression; decrease snail expression	A549 cells	—	0.005–0.01 g/L	Li (2015)
		Reduce Survivin expression; increase Caspase-3 expression	A549 cells	—	0.01 g/L	Li, 2015; Li et al., 2015; Sang, 2017
		Regulate Cdc42/MMP-2, MMP-9 pathway	A549 cells	—	5–10 μg/mL	Li et al. (2018b)
		Reduced F-actin formation	A549 cells	—	2.5 and 5 mg/L	Du, (2017)
		Inhibit the growth and metastasis of transplantation tumors; reduce the degree of bone destruction	BALB/c nude mice	i.g.	300 mg/kg	Sang, (2017)
	Dehydrocavidine	Inhibit hTERT expression	Tca8113 cells	—	0.050 and 0.100 g/L	Lei and Liao, (2008)
	CSB extract	Increase NF-κB P65 subunit expression	HepG2.2.15 cells	—	0.4, 0.8 and 1.6 mg/mL	Ju et al. (2018)
Anti-inflammatory and analgesic effects	CSB extract	Suppress oil-induced mice ear swelling; reduce painful torsional response in mice	KM mice	Transdermal administration	0.77 g/kg	Zhuge et al. (2019)
		Reduced serum TNF-α and IL-6 levels; suppress uterine swelling	Female Sprague Dawley rats	Rectal drug administration	4.2, 8.4 and 16.8 mg/kg	Xiao et al. (2019)
	CSBTA	Reduce TNF-α, IL-6 and CD86 expression; reduce IL-1β secretion	THP-1 cells	—	0.0025 and 0.005 g/L	Feng et al. (2020)
		Reduce TNF-α, IL-1β and PEG2 levels; inhibit p38 phosphorylation to block TRPV1 activation	Rats	p.o.	30, 60 and 120 mg/kg	Kuai et al. (2020)
		Inhibit RANKL-induced NF-κB and c-Fos/NFATc1 pathways; ameliorate cancer-induced osteolysis and bone pain	Female Wistar Han rats	i.g.	50 and 100 mg/kg	Ju et al. (2020)
			RAW 264.7 cells and MDA-MB-231 cells	—	50 μg/mL	
		Regulate PKCε/p38 MAPK/TRPV1 pathway; reduce pro-inflammatory cytokines and neuropeptides levels	Male Sprague Dawley rats	p.o.	30 and 120 mg/kg	Xue et al. (2021)
			DRG neuron cells of rat	—	50 μg/mL	
Antibacterial effects	CSBTA	Show inhibition of common Gram-positive and Gram-negative bacteria	—	—	MIC: 16.8–130 mg/mL	Qiu et al. (2020)
		Regulate the branched-chain amino acid, bile acid, arginine and proline, and purine metabolism	Male Sprague Dawley rats	p.o.	50 mg/kg	Liu et al. (2019a)
Antioxidant effects	Dehydrocavidine	Inhibition of oxidative stress damage; increase Bcl-2 expression; decrease Bax expression and ROS activity	MC3T3-E1 cells	—	0.1–10 μmol/L	Shi et al. (2020)
		Decrease SOD, GPx, CAT activity and increase MDA activity in the brain	Male Sprague Dawley rats	i.g.	50 mg/kg	Fu et al. (2018)

### Hepatoprotective Effects

The clearing away heat and detoxification of CSB is linked to its hepatoprotective effects. It demonstrated that CSB alleviated lesions of liver tissue and improved hepatic fibrosis through anti-oxidative stress, reducing live collagen deposition, inflammation and cell apoptosis.

Eighty Wistar rats were divided into six groups, including a normal group, a model group, a positive control group bifendate (150 mg/kg), and three CSBTA (from the CSB ethanol extract) groups (50, 75, and 100 mg/kg) (Liang et al., 2008). Except for the normal group, the animals in the other groups were injected subcutaneously with CCl_4_ (5 mL/kg 50% CCl_4_ for the first time, then used 3 mL/kg 30% CCl_4_) twice a week for modeling and it lasted for 12 weeks. Correspondingly, the rats in the normal group were given same volumes of peanut oil. Compared with the model, CSBTA (75 and 100 mg/kg) significantly reduced liver ALT, AST, Hyp, MDA, TGF-β1, and MMP-9 levels, and up-regulated decreased TP, ALB, SOD contents in the hepatic fibrosis rats. It was noted that the study only assessed the effect of simultaneous administration of CSBTA on CCl_4_-induced hepatic fibrosis. Actually, it is more reasonable to evaluate the effect of this extract from CSB after the successful establishment of CCl_4_-induced hepatic fibrosis model in the rats. In addition, the anti-hepatic fibrosis mechanism of CSB needs further clarification.

In addition, the effect of CSB on serum metabolomics of liver fibrosis was further explored (Tang and Guangxi, 2017). Forty SD rats were allocated into four groups: a normal, a model, a positive drug colchicines (0.1 mg/kg) and a CSB aqueous extract (2.5 g/kg). The 50% CCl_4_ olive oil solution was used for 10 weeks for modeling. The treatments were performed at weeks 7 and it lasted for four consecutive weeks. Serum was separated for NMR analysis after dosing cycle. Potential metabolic markers were selected after comparison with the two-dimensional HHCOSY map of NMR and the standard map of Human Metabolic Database. Finally the metabolic network was constructed with the help of KEGG and MetaboAnalyst 3.0 integrated mapping. The results showed that the intervention effect of CSB involved amino acid metabolism (leucine, valine, isoleucine, alanine, arginine, creatine), glucose metabolism (glucose, lactic acid, acetic acid), lipid metabolism (lipid, choline) and other metabolic pathways (nitroacetyl glycoprotein, oxyacetyl glycoprotein).

Wang et al. used dehydrocavidine, one of the high content constituents in CSBTA (about 40%), to preventively or curatively intervene SD rats with liver fibrosis induced by CCl_4_ (Wang et al., 2008). Dehydrocavidine (0.5 and 1 mg/kg) was intraperitoneally injected into the animals. The results showed that dehydrocavidine had no hepatotoxicity to healthy rats through analyzing serum biological, lipid peroxide and antioxidative, and histopathological parameters. Also dehydrocavidine above 0.5 mg/kg sigificantly decreased serum ALT, AST, ALP, TBIL and Hyp levels. In addition, before and after the modeling, dehydrocavidine markedly inhibited MDA product, GPx and SOD consumptions in the liver fibrosis rats, which was better than the positive drug glycyrrhizin (20 mg/kg). Subsequently, the urinary excretion of Hyp and the activity of CAT were determined (Wang et al., 2011). Also, they extracted the liver total RNA for microarray analyses to identify the fibrosis-related genes and then validated the results by real-time RT-PCR. It showed that 73 genes involving cell growth, proliferation, apoptosis, cytokines, transcription, and stress, were differentially expressed in the intoxicated rats compared with the control. Among them, four differential expressed genes (Bcl2, Cyp3a13, IL18, and Rad50) were validated. Finally, it was concluded that dehydrocavidine might act on these gene targets to against liver fibrosis.

Lu et al. obtained nine extracts from CSB by different extraction methods. MTT results indicated that each extract inhibited HSC-T6 cells proliferation activity at different levels (Lu et al., 2017). The potential active constituents in the extracts were identified by Scores plot and variable importance in projection values by means of orthogonal partial least squares analysis. Dehydrocavidine, palmatine and berberine were confirmed after screening. The antiproliferative activity of the three compounds ranging from 0.01 to 0.10 mg/mL was subsequently verified in the MTT assay and flow cytometry, respectively. The results showed that these three compounds inhibited proliferation and induced apoptosis in the cancer cells. Inhibition rate of palmatine and berberine at 0.10 mg/mL were higher than SB431542, the positive control drug. In addition, it suggested that the safe concentrations of palmatine and berberine were respectively less than or equal to 0.10 and 0.15 mg/mL.

Metabolomics and network pharmacology studies (Liu et al., 2018) revealed that CSB might achieve anti-hepatic fibrosis effects by intervening ALT, FXR, COX-2, MMP-1, AGT, GGT1, FHIT and GPD1 targets in rats. The potential active ingredients might be chelerthrine, sanguinarine, cavidine, dehydrocavidine and ferulamide. After reviewing the literatures, it was noticed that a monomer constituent named dehydrocavidine was potentially active among the total alkaloids.

An acute cholestasis rat model was established by α-naphthyl isothiocyanate-olive oil solution and then confirmed by pathological examination (Liu et al., 2019b). The protein expressions of NTCP, BSEP, MRP2 and MRP4 in liver tissue were detected by Western blot. The results showed that CSB water decoction up-regulated the expressions of BSEP and NTCP in the liver tissue, which suggested that CSB could regulate the intake and transfer of bile acids, improve the enterohepatic circulation of bile acid to treat or prevent early mild intrahepatic cholestasis. Interestingly, this study directly used the obtained solution also called “Tang-ji” after decocting CSB, which was consistent with the usage form of TCM. Although this study seems provide some explanations for the application of this ethnomedicine, it is necessary to discuss the pharmacodynamics difference between decoction and aqueous extract.

Chen et al. evaluated the effect of CSBTA on metabolic associated fatty liver disease (Chen et al., 2021). After 10 weeks feeding of high fat and sugar diet, C57BL/6 mice were randomly divided into various groups. The animals in the treatment groups were respectively given CSBTA (25 and 100 mg/kg) and metformin hydrochloride (200 mg/kg). The results showed that CSBTA significantly ameliorated liver hypertrophy and fatty lesions induced by high fat and sugar diet, including decreased serum TC, TG, LDL-C and NEFA levels. Compared with the model control, CSBTA and metformin significantly lowered fasting blood sugar and improved impaired glucose tolerance in the mice. Furthermore, CSBTA up-regulated *p*-AMPK, *p*-PI3K, and *p*-Akt protein expressions in the liver tissue, which might activate the AMPK/PI3K/Akt pathway blocked upon the high glucose environment.

It has been highlighted that CSB has inhibitory or killing effects on hepatitis B (Yin, 2001; Li et al., 2008; Zeng et al., 2013). Some studies (Li, 2010; Zhang et al., 2020) suggested that CSB rapidly produced antibodies *in vivo* and effectively stabilized hepatocyte membranes and mitochondrial membranes. Zeng et al. had evaluated the ability of dehydrocheilanthifoline to resist hepatitis B virus *in vitro* (Zeng et al., 2013). Dehydrocheilanthifoline effectively inhibited the secretion of HBsAg (IC_50_: 15.84 μM) and HBeAg (IC_50_: 17.12 μM), and reduce both intracellular and extracellular HBV DNA levels. Also, it promoted bile excretion and hepatocyte regeneration. An *in vivo* experiment (Wang et al., 2009) showed that CSB significantly inhibited duck hepatitis B virus (DHBA). Ten one-day-old Guangxi ducks received intraperitoneal injections of 0.2 ml DHBV-DNA positive virus serum. Seven days after the injections, positive infected ducks were selected by PCR and continuously fed until days 13. The animals were divided into six groups including a blank group, a model group, a positive drug group (acyclovir, 0.1 mg/kg), and three CSB groups (2, 4, 8 mg/kg). Compared with the model group, the high-dose CSB group (8 mg/kg) significantly reduced the serum DHBV-DNA, ALT and AST levels, while it was some contradictory to the results of a clinical trial (Li, 2010). The reason might be the clinical use of CSB at concentrations that did not achieve hepatitis B virus suppression. Therefore, it is necessary to evaluate the potential of CSB in clinic more acutely.

### Antitumor Effects

Currently, CSB exerts antitumor effects in tongue squamous cell carcinoma, lung cancer and liver cancer.

CSBTA (≤0.200 g/L) inhibited the proliferation and induced cell apoptosis of human tongue squamous cell carcinoma Tca8113, which might be associated with reduced telomerase activity by inhibiting NF-κB activation and Bcl2 expression inhibition at both mRNA and protein levels (Li et al., 2007; Lei and Liao, 2008; Xu and Liao, 2010; Yin and Liao, 2010; Zhu and Liao, 2011). However, whether Bcl2 functions as an upstream target of NF-κB pathway or a target of the apoptotic pathway is still unclear. Among the monomer constituents, dehydrocavidine has been confirmed to inhibit telomerase activity by inhibiting the expression of hTERT protein (Lei and Liao, 2008). Dehydroaporcavidine inhibited the activity of P50 and P65 subunits, thereby inhibiting the activation of NF-κB (Xu and Liao, 2010). And their inhibitory effects were better than CSBTA at the same concentration. Some issues were also present in this study, including the absence of the toxicology of CSB in the cancer cells and the positive control drug.

A series of studies showed that CSBTA inhibited the proliferation, migration, and induced apoptosis of non-small cell lung cancer A549 cells *in vitro* (Li, 2015; Li et al., 2015; Du, 2017; Sang, 2017; Li et al., 2018b). Flow cytometry showed that CSBTA arrested the cell cycle at phase G1 (Li, 2015). CSBTA ranging 0.005–0.1 g/L inhibited the cell proliferation. In addition, CSBTA displayed a similar inhibitory effect to cisplatin at 0.002 g/L at 48 h (Li et al., 2015). Flow cytometry also showed that CSBTA induced A549 cell apoptosis (Li, 2015; Li et al., 2015). RT-PCR results suggested that CSBTA at 0.01 g/L and cisplatin at 0.002 g/L down-regulated the mRNA level of Survivin, and up-regulated the Caspase-3 mRNA level. Although CSBTA inhibits proliferation, induces apoptosis and arrests the cell cycle, its specific mechanism needs further investigations.

Additionally, CSBTA might inhibit cancer cell migration by the following three pathways. First, CSBTA (0.005–0.01 g/L) increased the mRNA and protein expressions of E-cadherin, decreased the expression of snail, which might inhibit the EMT process (Li, 2015). Second, CSBTA (5–10 μg/mL) directly reduced both mRNA and protein levels of Cdc42, indirectly reduced its downstream factors MMP-2 and MMP-9 protein expressions in the cells, thereby inhibiting the migration and invasion of the A549 cells (Li et al., 2018b). Third, CSBTA reduced F-actin formation in the A549 cells, possibly enhanced Cofilin-1 activity by reducing Cofilin-1 phosphorylation (Du, 2017). After that, it was confirmed the proliferation inhibition in a nude mouse subcutaneous tumor model (Sang, 2017). After subcutaneous injections of A549 cell suspension, BALB/c nude mice were randomly divided into groups. The animals in the treatment groups were treated CSBTA (100 and 300 mg/kg) or cisplatin (2 mg/kg). Twenty-one days after the modeling, tumor volume and mass was determined. The results showed that both CSBTA and cisplatin inhibited the growth of the transplanted tumors. A bone metastasis model was also constructed to evaluate the antitumor effects of CSBTA by injecting the A549 cells into the left ventricle. Compared with the vehicle control group, CSBTA (300 mg/kg) and cisplatin slowed weight loss rate, reduced thoracic metastases, and reduced the serum BALP levels in the mice. These findings provided some *in vivo* evidences for CSBTA in the treatment of lung cancer. However, the positive control drug cisplatin is not reasonable and bisphosphonate may be a better choice.

In an early experiment *in vitro*, dehydrocavidine had no significantly inhibitory effect on human liver cancer cell lines HepG2 and QCY-7703 (Huang, 2015). However, a recent study found that CSB water extract inhibited the proliferation and migration of the HepG2 cells, and up-regulated the intracellular expression of NF-κB P65 subunit (Ju et al., 2018). Thus, it is encouraged to confirm the active constituents in the aqueous extract for the treatment of hepatic cancer.

### Anti-inflammatory and Analgesic Effects

The anti-inflammatory effects support its relieving pain. Intraperitoneal administration of CSBTA (50 mg/kg) reduced paw edema in rats with arthritis induced by egg white injection, while it had no significant effect in rats with formaldehyde arthritis at the same dose (Huang et al., 1981). The CSB injection significantly inhibited xylene-induced ear swellings in mice at the early stage of inflammation (Li, 2009). CSB rectal suppository had the similar effect on treating croton oil-induced mice ear swelling (Zhuge et al., 2019). Meanwhile, CSB at 0.4375 mg/kg inhibited the formation of cotton ball granuloma in mice at late inflammation. Also, CSB rectal suppository showed good analgesic and anti-inflammatory effects *in vivo*. Xiao et al. found that the CSB suppository (0.77 g/kg) significantly reduced the serum TNF-α and IL-6 levels in rats with pelvic inflammatory disease, and the effect was approximately equivalent to levofloxacin (Xiao et al., 2019). On the basis of the inhibition of inflammatory factor production, a subsequent study suggested that CSBTA might improve the inflammatory environment *via* effectively suppressing M1 polarization of THP-1-derived macrophages (Feng et al., 2020).

For peripheral neuropathy, CSBTA also showed good anti-inflammatory and analgesic properties both *in vivo* and *in vitro*. Kuai et al. evaluated cisplatin-induced peripheral neuropathy in rats. The results showed that CSBTA (30, 60 and 120 mg/kg) by oral administration significantly reduce pain symptoms together with decreased levels of pro-inflammatory cytokine such as TNF-α, IL-1β and PGE2 (Kuai et al., 2020). Importantly, it improved intraepidermal nerve fiber loss and inhibited inflammation-induced p38 phosphorylation to block TRPV1 activation. Xue et al. evaluated paclitaxel-induced peripheral neuropathy in rats and DRG neuron cells of rats (Xue et al., 2021). *In vivo*, CSBTA (30 and 120 mg/kg) by oral administration reduced TNF-α, IL-1β, PGE2, CGRP and SP levels. CSBTA at 120 mg/kg effectively reduce PKCε, p-p38, MAPK and TRPV1 protein expressions and mRNA levels. The similar effects *in vitro* required 50 μg/mL of CSBTA. These two studies showed that CSBTA achieves anti-inflammatory and analgesic effects by inhibiting p38 phosphorylation and blocking TRPV1 activation. PKCε is one of the upstream targets of this pathway. However, positive control drugs were missing in these experiments.

In addition, Ju et al. constructed a cancer bone pain model by intraperitoneal injection of 0.5 mL Walker 256 cell suspension into Wistar rats. *In vivo*, CSBTA significantly alleviated bone pain in rats without obvious adverse effects at doses of 50 and 100 mg/kg. *In vitro* CSBTA at 50 μg/mL inhibited osteoclastogenesis by inhibiting RANKL-induced NF-κB and c-Fos/NFATc1 pathways (Ju et al., 2020). Overall, CSB achieves its anti-inflammatory and analgesic effects mainly by affecting the production of pro-inflammatory cytokines and regulating related inflammatory pathways.

### Antibacterial Effects

CSBTA showed inhibitory effect (MIC: 16.8–130 mg/mL) against common Gram-positive and Gram-negative bacteria *in vitro* (Qiu et al., 2020). Except for *Candida albicans* and *Pseudomonas aeruginosa* (MBC>300 mg/mL), it had a certain bactericidal effect on *Staphylococcus aureus*, *Streptococcus pyogenes*, *Streptococcus faecalis*, *Escherichia coli*, *Helicobacter flexneri*, *Klebsiella pneumoniae*, *Salmonella typhi*, *Salmonella enteritidis* and *Proteus*. Sun et al. determined the antibacterial effect of CSB aqueous extract combined with penicillin, cefradine, fosfomycin and levofloxacin respectively on *S.aureus* (Sun et al., 2020). The results showed that CSB had a synergistic effect when combined with cefradine, penicillin, and levofloxacin (FIC≦0.5). However, unreasonable combination of medicine may enhance toxicity and increase adverse drug reactions. The toxicity of the CSB extract alone or combined with antibiotics need clarification to support its safety in clinical application.

*In vivo*, Liu et al. investigated the effect of CSBTA on antibiotic-induced gut microbiota dysbiosis (Liu et al., 2019a). After rats received gavage administration of imipenem/cilastatin sodium (50 mg/kg), ten genera were found to be disturbed. But CSBTA at the same dose by oral administration restored four genera of them, especially *g_Blautia*. The metabolomic results indicate that CSBTA regulates the imbalanced microbiota in the gut mainly through the metabolism of branched-chain amino acid, bile acid, arginine and proline, and purine. Although there are few studies on the antimicrobial properties of CSB, its potential is still worth exploring in this field.

### Antioxidant Effects

CSB exhibited antioxidant activity in some *in vivo* and *in vitro* experiments. He et al. extracted the whole CSB plant using 70% ethanol and isolated and identified 16 compounds after silica gel column chromatography analysis and spectroscopic analysis (He et al., 2014). In DPPH radical scavenging experiment, CSBTA showed strong antioxidant activity, especially cheilanthifoline (IC_50_: 0.25 mg/mL) and isocorydine (IC_50_: 1.00 mg/mL). Subsequently, in a MC3T3-E1 cell injury model induced by H_2_O_2_ (500 μmol/L) (Shi et al., 2020), MTT result showed that dehydrocavidine ranging 0.001–10 μmol/L had no significant effect on the cell survival. Compared with the model, dehydrocavidine above 0.1 μmol/L or N-acetylcysteine (1 mmol/L) remarkably inhibited the oxidative stress injury, including reduced apoptosis, increased Bcl-2 expression, decreased Bax expression and ROS activity.

The antioxidative effect of CSB has also been confirmed in liver disease models. In addition, dehydrocavidine improved learning and memory impairment induced by d-gala in rats through reducing oxidative damage (Fu et al., 2018). The degree of learning and memory impairment in rats was reduced after 8 weeks of gavage administration of dehydrocavidine at 50 mg/kg. Meanwhile dehydrocavidine decreased SOD, GPx, CAT activity and increased MDA activity in the brain. Interestingly, dehydrocavidine at the same dose did not affect the normal rats, which suggested a low toxicity of this constituent in the brain. However, the positive control drug is absent in this study.

## Applications

Generally, CSB is called “Yan-huang-lian” in Chinese. Also, it is called “Yan-hu” (Guizhou), “Tu-huang-lian” (Guangxi), “Yan-lian” (Sichuan and Yunnan) (Chinese Materia Medica Editorial Committee, 1999). CSB is bitter and cool in taste. Traditionally, CSB has been use to clear away heat and detoxicate, remove dampness, relieve pain and hemostasis. It has been used to treat acute conjunctivitis (called “huo-yan”), corneal pannus, acute abdominal pain, hemorrhoidal bleeding, haematochezia, swelling, hepatitis, cirrhosis and liver cancer (Jiangsu New Medical College, 1986; Guangxi Zhuang Autonomous Region Department of Health, 1992) (Table 4).

**TABLE 4:** The application of CSB.

Component(s)	Traditional uses	Usage	Reference
CSB 5 g, *Radlx gentianae* 5 g, borneol 0.01 g	Curing acute conjunctivitis and corneal pannus	Grind into powder, steam and apply to the eyes	Jiangsu New Medical College, (1986)
CSB 5 g	Curing hemorrhoidal bleeding and haematochezia	Steam with wine and take orally (100 g)	Jiangsu New Medical College, (1986)
CSB 10 g	Curing acute abdominal pain	Take orally	Jiangsu New Medical College, (1986)
CSB 3–15 g	Curing hepatitis	Take orally	Chinese Materia Medica Editorial Committee, (1999)

Nowadays, the preparations of CSB mainly include injections, tablets and suppositories. Only the injection preparation is used in clinical practice at present (Luo, 2009). However, its safety is controversial. Given the demonstrated oral effectiveness of CSB, CSBTA capsules are in clinical trials. Capsules may become the primary form for clinical application of this ethnomedicine in the future.

## Toxicology

Currently, there are few complete toxicological studies on CSB. Huang et al. conducted acute and long-term toxicity tests on the CSB extract (Huang et al., 2007). Fifty healthy KM mice were divided into five groups including various doses of CSB at 560, 450, 360, 290, and 230 mg/kg. The animals were administered for three times within 24 h and observed for seven consecutive days. The results showed that the LD_50_ of the CSB extract was 298.5 mg/kg (95% CI: 257.2–346.5 mg/kg). The long-term toxicity also indicated that the toxicity of the CSB extract was relatively small. However, the details for the experimental process were not mentioned in the study.

Sun evaluated the preclinical safety of the dehydrocavidine injection, including safety pharmacology, acute toxicity, long-term toxicity, allergic, irritation, hemolysis and other toxicities (Sun, 2007). In the safety pharmacology study, intravenous dehydrocavidine ranging from 0.2 to 0.8 mg/kg had no adverse effect on the cardiovascular and respiratory systems of beagle dogs. Intravenous administration of dehydrocavidine had no adverse effect on locomotor activity and pole climbing ability of KM mice. In the acute toxicity test, the intravenous MTD of dehydrocavidine were above 40 and 20 mg/kg in mice and rats, respectively. The intraperitoneal MTD were above 50 and 30 mg/kg in mice and rats. In the long-term toxicity test, after intravenous injection of dehydrocavidine (0.25–2.50 mg/kg) for 180 days in beagle dogs, the animals developed movements such as scratching and salivation after the administration of high-dose-dehydrocavidine, while it was recovered after the drug withdrawal. The safe dose of intravenous dehydrocavidine in the beagle dogs was 0.75 mg/kg, and the toxic dose was 2.50 mg/kg. In addition, other toxicity tests showed that dehydrocavidine above the clinical dose did not cause irritation and adverse damage in rabbits and guinea pigs. Therefore, dehydrocavidine showed high safety in the experimental animals. And the toxic reactions caused by overdose in the beagle dogs were mild and reversible. However, this conclusion is not suitable for CSBTA, for dehydrocavidine just one constituent of it.

In some recent *in vitro* pharmacological studies, researchers have found that high doses of CSBTA (50 μg/mL) was not cytotoxic to a variety of cell lines (Ju et al., 2020; Xue et al., 2021). In addition, CSBTA was found to inhibit cytochromes P1A2 (IC_50_: 38.08 μg/mL), P2D1 (IC_50_: 20.89 μg/mL), P2C6/11 (IC_50_ for diclofenac and S-mephenytoin: 56.98 and 31.59 μg/mL), and P2B1 (IC_50_: 48.49 μg/mL) (Yu et al., 2018). Therefore there is a risk when CSBTA is combined with drugs metabolized by these cytochromes.

Toxicological studies of other constituents in CSBTA also seem to provide some insight into the safety of the drug. The study found that berberine, a constituent of CSBTA, generally was considered safe at clinical doses (Bansod et al., 2021). However, gastrointestinal discomfort, reduced blood pressure, heart damage, shortness of breath and flu-like symptoms might occur at high doses. Besides, berberine exhibited some phototoxicity (Singh et al., 2021). Of concern is that palmatine, another major constituent of CSBTA, exhibited toxicity to a variety of cell lines. Also it produced damage to DNA through oxidative stress (Long et al., 2019). In summary, the safety of CSB needs further assessments.

## Conclusion

CSB is a commonly ethnomedicine in Southwest China and it has a long history of use in the Chinese folk. In the present study the phytochemistry, pharmacology, applications and toxicology of CSB have been reviewed. Consistent with traditional use, the protective effect of CSB on the liver has been widely recognized. In addition, CSB also has a variety of pharmacological activities such as anti-inflammatory, antioxidant and anticancer activities.

There are three main types of drug sources in the current studies on CSB: *1*) CSBTA; *2*) CSB extract; *3*) monomer constituent (such as dehydrocavidine). Due to the unstable content of compounds in plants from different regions and the differences in chemical composition between wild and cultivated products, it is difficult to accurately assess the therapeutic concentration of this drug. Thus, establishing quality standards for this medicinal herb is urgent. Furthermore exploring purification methods for CSBTA monomer constituents is encouraged.

Kinetic studies have shown that the main sites of metabolism of CSBTA are the liver and intestine. Except for few common alkaloids such as dehydrocavidine, pharmacodynamics of other active ingredients is also required. In addition, toxicological studies need to be strengthened as well to support their therapeutic safety.

At present, the pharmacological studies of CSB are still in the stage of identifying active ingredients. So, pharmacological studies should be addressed as follows: *1*) the main mechanisms by which CSBTA or other monomeric compounds exert their pharmacological effects; *2*) methods to achieve the same efficacy in animal models; *3*) evaluating safety and efficacy of the therapeutically potential reagents in clinical trials. In this review, non-hepatic chronic diseases and tumours may be new research directions for this plant. For example, based on the metabolic characteristics of it and pharmacological activity of alkaloid constituents, it suggests the possibility of CSB in intervening intestinal tumors.

In this review, we summarized and analyzed the traditional uses, phytochemistry, pharmacokinetics, pharmacology, toxicity, and applications of *Corydalis saxicola* Bunting. The issues in the research and development of this plant were proposed as well as the solutions. Further, some new research directions such as anti-tumor, anti-inflammatory related diseases, and analgesia were also provided to utilizing this ethnomedicine more deeply in future.

## Glossary

AGT: angiotensinogen
Akt: protein kinase B
ALB: albumin
ALP: alkaline phosphatase
ALT: alanine aminotransferase
AMPK: adenosine 5’-monophosphate-activated protein kinase
AST: aspartate aminotransferase
Bax: Bcl2-associated x
Bcl2: B-cell lymphoma-2
BSEP: bile salt export pump
CAT: catalase
CCl4: carbon tetrachloride
Cdc42: cell division cycle 42
COX-2: cyclooxygenase-2
CSB: Coradalis Saxicola Bunting
CSBTA: Coradalis Saxicola Bunting total alkaloids
CYP: cytochrome P450
DAD: diode array detection
DHBV: duck hepatitis B virus
DRG: dorsal root ganglion
EMT: epithelial-mesenchymal transition
FHIT: fragile histidine triad
FIC: fractional inhibitory concentration
FXR: farnesoid X receptor
GGT1: gamma-glutamyltransferase 1
GPD1: glycerol-3-phosphate dehydrogenase 1
GPx: glutathione peroxidase
HHCOSY: 1H-1H correlation spectroscopy
HPLC: high-performance liquid chromatography
hTERT: human telomerase reverse transcriptase
Hyp: hydoxyproline
IL-6/18/1β: interleukin-6/18/1β
KEGG: kyoto encyclopedia of genes and genomes
LDL-C: low density lipoprotein-cholesterol
MBC: minimum bactericidal concentration
MDA: malondialdehyde
MIC: minimum inhibitory concentration
MMP-1/2/9: matrix metallopeptidase-1/2/9
MRP2/4: multidrug resistance-associated protein 2/4
MTD: maximum tolerated dose
MTT: 3-(4,5-Dimethylthiazol-2-yl)-2,5-diphenyltetrazolium bromide
NEFA: nonestesterified fatty acid
NFATC1: nuclear factor-activated T cell 1
NF-κB: nuclear factor kappa-B
NMR: nuclear magnetic resonance
NTCP: sodium taurocholate cotransporting polypeptide
PGE2: prostaglandin E2
PI3K: phosphatidylinositol three kinase
PKCε: protein kinase ε
RANKL: Receptor activator of nuclear factor-κ B ligand
RT-PCR: reverse transcription-polymerase chain reaction
SOD: superoxide dismutase
SP: substance P
TBIL: total bilirubin
TC: total cholesterol
TCM: traditional Chinese medicine
TG: triglyceride
TGF-β1: transforming growth factor-β1
THP-1: human myeloid leukemia mononuclear cells
TNF-α: tumor necrosis factor-α
TP: total protein
TRPV1: transient receptor potential vanilloid 1

## References

[B1] Bansod S. Saifi M. A. Godugu C. (2021). Molecular Updates on Berberine in Liver Diseases: Bench to Bedside. Phytother Res. 35, 5459–5476. 10.1002/ptr.7181 34056769

[B2] Chen P. Ju L. J. Chen J. Diao H. F. Zhao K. J. Qiu Z. X. (2021). Study on Effect and Molecular Mechanism of Corydalis Saxicola Total Alkaloids on Nonalcoholic Fatty Liver Mice. Drug Eval. Res. 44, 468–477. 10.19378/j.issn.1003-9783.2018.01.021

[B3] Cheng X. Wang D. Jiang L. Yang D. (2008a). DNA Topoisomerase I Inhibitory Alkaloids from Corydalis Saxicola. Chem. Biodivers 5, 1335–1344. 10.1002/cbdv.200890121 18649321

[B4] Cheng X. Wang D. Jiang L. Yang D. (2008b). Simultaneous Determination of Eight Bioactive Alkaloids in Corydalis Saxicola by High-Performance Liquid Chromatography Coupled with Diode Array Detection. Phytochem. Anal. 19, 420–428. 10.1002/pca.1067 18446771

[B5] Chinese Materia Medica Editorial Committee (1999). Chinese Materia Medica. Shanghai: Shanghai Scientific & Technical Publishers.

[B6] Cicero A. F. Baggioni A. (2016). Berberine and its Role in Chronic Disease. Adv. Exp. Med. Biol. 928, 27–45. 10.1007/978-3-319-41334-1_2 27671811

[B7] Cicero A. Ertek S. (2009). Berberine: Metabolic and Cardiovascular Effects in Preclinical and Clinical Trials. Nutr. Dietary Supplements Vol. 1, 1–10. 10.2147/NDS.S6084

[B8] Dai G. Sun B. Wu L. Gao X. Song S. Sun H. (2018). Comparative Pharmacokinetics of Three Alkaloids in Normal and Acute Hepatitis Rats After Oral Administration of Yanhuanglian Total Alkaloids Extract. Biomed. Chromatogr. 32, e4329. 10.1002/bmc.4329 29972688

[B9] Deng J. Xiao X. Li G. Ruan G. (2009). Application of Microwave-Assisted Extraction Coupled with High-Speed Counter-current Chromatography for Separation and Purification of Dehydrocavidine from Corydalis Saxicola Bunting. Phytochem. Anal. 20, 498–502. 10.1002/pca.1152 19742782

[B10] Du Y. J. (2017). Corydalis Saxicola Bunting Extractive Inhibit F-Actin Polymerization in A549 Cells and its Possible Mechanism. Guangxi, China: Guilin Medical University.

[B11] Feng C. Yong X. Z. Jiang Q. Z. Wu T. T. Jiang L. L. Su Z. H. (2020). Inhibitory Effects of Corydalis Saxicola Bunting Total Alkaloids on M1 Macrophages Polarization. J. Guangxi Med. Univ. 37, 2103–2110. 10.16190/j.cnki.45-1211/r.2020.12.002

[B12] Fu P. Zhang Q. Yi D. Y. Abdelmaksoud A. Huang Q. Zhao H. Y. (2018). Dehydrocavidine Attenuates D-Galactose Induced Learning and Memory Impairment in Rats. Neurosci. Lett. 665, 200–205. 10.1016/j.neulet.2017.12.004 29208407

[B13] Guangxi Zhuang Autonomous Region Department of Health (1992). Guangxi Traditional Chinese Medicine Standard 1990 Edition. Nanning: Guangxi Sci. & Technol. Publishing House.

[B14] He Z. C. Wang D. M. Li G. C. Wu J. Y. (2014). Study on Alkaloids from Corydalis Saxicola and Their Anti-oxidative Activities. China. Tradit. Herb. Drugs 45, 1526–1531. 10.7501/j.issn.0253-2670.2014.11.005

[B15] Huang P. (2015). Synergistic Effects and Decreasing Toxicity on the Fufang Yanzhang Capsule for Mouse Sarcoma and its Main Components Inhibiting the Proliferation of Hepatocellular Carcinoma Cells. Guangxi Med. Univ.

[B16] Huang X. N. Liu G. X. Zhang X. D. (1981). Preliminary Observation on the Analgesic, Cholagogue and Cholagogue Effects of Corydalis Saxicola Total Alkaloids. J. Zunyi Med. Univ. 4, 22–25.

[B17] Huang X. Z. Liu X. P. Huang M. Huang Z. Q. Li D. Jiang W. Z. (2007). Study on Main Pharmacodynamics and Acute Toxicity of Herba Corydalis Saxicolae Extract. Chin. J. Hosp. Pharm. 27, 146–148.

[B18] Huang Q. Q. Bi J. L. Sun Q. Y. Yang F. M. Wang Y. H. Tang G. H. (2012). Bioactive Isoquinoline Alkaloids from Corydalis Saxicola. Planta Med. 78, 65–70. 10.1055/s-0031-1280126 21858757

[B19] JiangsuNew Medical College (1986). Chinese Medicine Dictionary. Shanghai: Shanghai Scientific & Technical Publishers.

[B20] Ju J. N. Ming Z. Y. Dai Q. K. Li K. Z. Huang S. He J. B. (2018). Effect of Corydalis Saxicola Bunting Aqueous Extract on Proliferation and Migration of Hepatocellular Carcinoma HepG2 Cells and its Possible Mechanism. Chin. J. Oncol. Prev. Treat. 10, 434–438. 10.3969/j.issn.1674-5671.2018.06.03

[B21] Ju L. Hu P. Chen P. Wu J. Li Z. Qiu Z. (2020). Corydalis Saxicola Bunting Total Alkaloids Attenuate Walker 256-Induced Bone Pain and Osteoclastogenesis by Suppressing RANKL-Induced NF-Κb and C-Fos/NFATc1 Pathways in Rats. Front. Pharmacol. 11, 609119. 10.3389/fphar.2020.609119 33574755 PMC7870471

[B22] Ke M. M. Zhang X. D. Wu L. Z. Zhao Y. Zhu D. Y. Song C. Q. (1982). Study on Active Ingredients of Corydalis Saxicola Bunting. J. Integr. Plant Biol. 24, 289–291.

[B23] Kuai C. P. Ju L. J. Hu P. P. Huang F. (2020). Corydalis Saxicola Alkaloids Attenuate Cisplatin-Induced Neuropathic Pain by Reducing Loss of IENF and Blocking TRPV1 Activation. Am. J. Chin. Med. 48, 407–428. 10.1142/S0192415X20500214 32138533

[B24] Kukula-Koch W. A. Widelski J. (2017). “Alkaloids,” in Chapter 9 - Alkaloids" in Pharmacognosy. Editors Badal S. Delgoda R. (Boston, MA: Academic Press), 163–198. 10.1016/b978-0-12-802104-0.00009-3

[B25] Lei J. Liao J. X. (2008). Tetradehydroscoulerine on Cell Proliferation and hTERT Expression of Tca8113 Cell Lines. J. Oral Maxillofac. Surg. 18, 173–177.

[B26] Li L. (2009). Experimental Study on the Anti-inflammatory Activity of Yanhuanglian. Chin. J. Ethnomedicine Ethnopharmacy 18, 20–21. 10.1088/1674-1056/18/10/033

[B27] Li S. X. (2010). Clinical Observation on the Treatment of Viral Hepatitis with Corydalis Saxicola Bunting. China Foreign Med. Treat. 29, 104. 10.16662/j.cnki.1674-0742.2010.01.018

[B28] Li J. H. (2015). The Effect of Corydalis Saxibola Bunting Total Alkaloids for Lung Cancer A549 Cell and its Possible Mechanism. Guangxi, China: Guilin Medical University.

[B29] Li H. L. Zhang W. D. Zhang C. Liu R. H. Wang X. W. Wang X. L. (2006). Bioavailabilty and Pharmacokinetics of Four Active Alkaloids of Traditional Chinese Medicine Yanhuanglian in Rats Following Intravenous and Oral Administration. J. Pharm. Biomed. Anal. 41, 1342–1346. 10.1016/j.jpba.2006.03.029 16644173

[B30] Li J. F. Liao J. X. Li H. L. Zhang W. D. (2007). Inhibition of Yanhuanglian Total Alkaloids on Cell Proliferation and Telomerase Activity of Tca8113 Cell Lines. J. Oral Maxillofac. Surg. 19, 32–35.

[B31] Li H. L. Han T. Liu R. H. Zhang C. Chen H. S. Zhang W. D. (2008). Alkaloids from Corydalis Saxicola and Their Anti-hepatitis B Virus Activity. Chem. Biodivers 5, 777–783. 10.1002/cbdv.200890074 18493964

[B32] Li J. H. Wang J. Y. Zeng J. R. Gao Y. Li M. M. Yu Y. Y. (2015). Effect of Corydalis Saxicola Total Alkaloids on Human A549 Cell Proliferation, Apoptosis and Expressions of Caspase, Survivin. Chin. J. Exp. Traditional Med. Formulae 21, 165–169. 10.13422/j.cnki.syfjx.2015090165

[B33] Li K. Zhang D. J. Li Z. Q. Lu D. X. (2018a). Alkaloids in the Corydalis Plants and Their Biological Activities: Research Advances. J. Int. Pharm. Res. 45, 748–757. 10.13220/j.cnki.jipr.2018.10.004

[B34] Li M. Wang J. Mo B. Zeng J. Yao D. Chen F. (2018b). Total Alkaloids of Corydalis Saxicola Bunting Inhibits Migration of A549 Cells by Suppressing Cdc42 or Vav1. Oncol. Lett. 15, 475–482. 10.3892/ol.2017.7273 29285198 PMC5738710

[B35] Liang Y. H. Jia J. Spencer P. S. Mao Y. A. Qin C. J. (2008). Protective Effect of Corydalis Saxicola Alkaloids(CSA) on Level of TGF-Β1, MMP-9 in Rats with Liver Fibrosis. Lishizhen Med. Materia Med. Res. 19, 2620–2622.

[B36] Liang Y. H. Tang C. L. Lu S. Y. Cheng B. Wu F. Chen Z. N. (2016). Serum Metabonomics Study of the Hepatoprotective Effect of Corydalis Saxicola Bunting on Carbon Tetrachloride-Induced Acute Hepatotoxicity in Rats by (1)H NMR Analysis. J. Pharm. Biomed. Anal. 129, 70–79. 10.1016/j.jpba.2016.06.033 27399344

[B37] Liu X. Feng J. Jin C. Chen M. (2009). Absorption Kinetics of Dehydrocavidine in Rats' Stomachs and Intestines. Zhongguo Zhong Yao Za Zhi 34, 1022–1026. 19639793

[B38] Liu X. W. Tang C. L. Zheng H. Wu J. X. Wu F. Mo Y. Y. (2018). Investigation of the Hepatoprotective Effect of Corydalis Saxicola Bunting on Carbon Tetrachloride-Induced Liver Fibrosis in Rats by 1H-NMR-Based Metabonomics and Network Pharmacology Approaches. J. Pharm. Biomed. Anal. 159, 252–261. 10.1016/j.jpba.2018.06.065 29990893

[B39] Liu X. Zheng H. Lu R. Huang H. Zhu H. Yin C. (2019a). Intervening Effects of Total Alkaloids of Corydalis Saxicola Bunting on Rats with Antibiotic-Induced Gut Microbiota Dysbiosis Based on 16S rRNA Gene Sequencing and Untargeted Metabolomics Analyses. Front. Microbiol. 10, 1151. 10.3389/fmicb.2019.01151 31214133 PMC6555270

[B40] Liu X. Z. Qiu J. N. Qi L. Sang N. N. Luo S. B. Liu J. (2019b). Effects of Corydalis Saxicola Bunting on Acute Cholestasis in Rats and Expression of Related Bile Acid Transporter. China J. Traditional Chin. Med. Pharm. 34, 1700–1703.

[B41] Long J. Song J. Zhong L. Liao Y. Liu L. Li X. (2019). Palmatine: A Review of its Pharmacology, Toxicity and Pharmacokinetics. Biochimie 162, 176–184. 10.1016/j.biochi.2019.04.008 31051209

[B42] Lu S. Y. Zheng H. Cheng B. Wu F. Wu J. X. Liu X. W. (2017). Discrimiation of Proliferation Inhibiting Ingredients in Corydalis Saxicola on Rat Hepatic Stellate Cell-T6 Based on Composition-Activity Relationship. Chin. Traditional Herbal Drugs 48, 1354–1361. 10.7501/j.issn.0253-2670.2017.07.016

[B43] Luo Y. X. (2009). Studies on Quality of Corydalis Saxicola Bunting and its Preparations. Chin. J. Pharm. Anal. 29, 1976–1980.

[B44] Mao Y. A. (2006). Chemical Constituents and Activity of Corydalis Saxicola Bunting. Guangxi, China: Guilin Medical University.

[B45] Qiu Q. Q. Wei Z. P. Wu L. M. Liu Y. W. Meng T. X. (2020). Experimental Study on Antibacterial Activity of Chinese Herb Yanhuanglian In Vitro. Sci. Tech. Innovation, 43–44. 10.15913/j.cnki.kjycx.2020.09.015

[B46] Sang Y. (2017). Research of Corydalis Saxicola Bunting Total Alkaloids on Nude Mouse with Subcutaneous Transplantation Tumor and Bone Metastasis in Lung Cancer A549. Guangxi, China: Guilin Medical University.

[B47] Shi Y. J. Xie H. Mao C. Q. Zhang X. D. Zheng X. P. (2013). Intestinal Transport Characteristics of Dehydrocavidine. Chin. Traditional Patent Med. 35, 256–260.

[B48] Shi Y. H. Huang G. Y. Xue Z. Y. Chen J. W. Zhu J. H. Wu J. Y. (2020). The Effect of Dehydrocavidine on the Proliferation and Apoptosis of Cells MC3T3-E1 Induced by H2O2. J. Chin. Med. Mater. 43, 457–463. 10.13863/j.issn1001-4454.2020.02.038

[B49] Singh S. Pathak N. Fatima E. Negi A. S. (2021). Plant Isoquinoline Alkaloids: Advances in the Chemistry and Biology of Berberine. Eur. J. Med. Chem. 226, 113839. 10.1016/j.ejmech.2021.113839 34536668

[B50] Sun N. L. (2007). Pharmacodynamic Studies and Preclinical Safety Evaluatons of Dehydrocavidine. Beijing, China: Academy of Military Sciences.

[B51] Sun T. T. Jiang P. Zhong Z. M. Fang Y. Yang Y. Q. Shan H. H. (2020). Bacteriostasis of Corydalis Saxicola Combined with Antibiotics on staphylococcus Aureus In Vitro. J. Jinggangshan University(Natural Science) 41, 103–106.

[B52] Tang C. L. (2017). The Study of Establishing the Fingerprint Chromatogram and Revealing the Anti-hepatic Fibrosis Effect of Corydalis Saxicola Bunting Based on Serum Metabonomics. Guangxi, China: Guilin Medical University.

[B75] Tang C. L. Liu P. Zheng H. Song H. Wang J. Liang Y. H. (2018). The Chemical Constituents and Pharmacological Effects of Corydalis saxicola Bunting: A Review. Tradit. Chinese Drug Res. Clin. Pharmacol. 29, 104–109. 10.19378/j.issn.1003-9783.2018.01.021

[B53] Tang C. L. Yang H. H. Huang X. M. Shen Y. D. Huang Y. H. Song H. (2019). Quality Assessment of Corydalis Saxicola Bunting Based on HPLC Fingerprint and Multi-Components Quantitative Determination. China J. Traditional Chin. Med. Pharm. 34, 100–104.

[B54] Wang Q. Z. Liang J. Y. Yuan Y. (2007). Chemical Constituents of Corydalis Saxicola. Chin. J. Nat. Medicines 5, 31–34.

[B55] Wang T. Sun N. L. Zhang W. D. Li H. L. Lu G. C. Yuan B. J. (2008). Protective Effects of Dehydrocavidine on Carbon Tetrachloride-Induced Acute Hepatotoxicity in Rats. J. Ethnopharmacol 117, 300–308. 10.1016/j.jep.2008.02.010 18358653

[B56] Wang J. Zhang S. J. Wu S. H. Jiang W. Z. (2009). Inhibitory Effect of Extract from Corydalis Saxicola Bunting on HBV In Vivo. China Pharmaceuticals 18, 7–9.

[B57] Wang T. Zhao L. J. Li P. Jiang H. Lu G. C. Zhang W. D. (2011). Hepatoprotective Effects and Mechanisms of Dehydrocavidine in Rats with Carbon Tetrachloride-Induced Hepatic Fibrosis. J. Ethnopharmacol 138, 76–84. 10.1016/j.jep.2011.08.039 21893185

[B58] Wu Y. R. Ma Y. B. Zhao Y. X. Yao S. Y. Zhou J. Zhou Y. (2007). Two New Quaternary Alkaloids and Anti-hepatitis B Virus Active Constituents from Corydalis Saxicola. Planta Med. 73, 787–791. 10.1055/s-2007-981549 17611928

[B59] Wu Y. Lu T. L. Ji D. Zhou Y. Mao C. Q. (2015). Isolation and Structural Identification of Alkaloids from Corydalis Saxicola. J. Nanjing Univ. Traditional Chin. Med. 31, 81–83. 10.14148/j.issn.1672-0482.2015.0081

[B60] Wu F. Liu X. Liang Y. H. Zheng H. Su Z. H. Song H. (2021). MDF-based Metabolites of Aqueous Extract of Corydalis Saxicola Bunting in Rats' Plasma, Urine, Bile and Stool. Lishizhen Med. Materia Med. Res. 32, 28–35.

[B61] Xia J. Z. (2002). Comparison of TLC Between Corydalis Saxicola Bunting and Coptidis Rhizoma. Res. Pract. Chin. Medicines 36. 10.2753/csh0009-4633360181

[B62] Xiao P. Lin C. X. Pan B. J. Zhuge M. L. Huang Y. Zeng D. Y. (2019). Pharmacodynamics of Yanhuanglian Suppository for Rats with Chronic Pelvic Inflammatory Diseases. Cent. South Pharm. 17, 2052–2058.

[B63] Xie G. Jin S. Li H. Ai M. Han F. Dai Y. (2021). Chemical Constituents and Antioxidative, Anti-inflammatory and Anti-proliferative Activities of Wild and Cultivated Corydalis Saxicola. Ind. Crops Prod. 169, 113647. 10.1016/j.indcrop.2021.113647

[B64] Xu K. Yuan X. L. Li C. Li A. X. (2020). Recent Discovery of Heterocyclic Alkaloids from Marine-Derived Aspergillus Species. Mar. Drugs 18, 54. 10.3390/md18010054 PMC7024353 31947564

[B65] Xu R. Liao J. X. (2010). Effects of Yanhuanglian Total Alkaloids and Dehydroapocavidine on NF-Kappa B Actiity of Oral Carcinoma Cell Lines. J. Oral Maxillofac. Surg. 20, 241–244.

[B66] Xue C. Liu S. X. Hu J. Huang J. Liu H. M. Qiu Z. X. (2021). Corydalis Saxicola Bunting Total Alkaloids Attenuate Paclitaxel-Induced Peripheral Neuropathy through PKCε/p38 MAPK/TRPV1 Signaling Pathway. Chin. Med. 16, 58. 10.1186/s13020-021-00468-5 34281577 PMC8287815

[B67] Yin H. (2001). Effect of Yanhuanglian and Danshen Injection on Liver Fibrosis of Chronic Hepatitis B. J. Pract. Med. 17, 782–783.

[B74] Yin J. K. Liao J. X. (2010). Effects of Corydalis saxicola Bunting Total Alkaloids on Tca8113 Cell Proliferation and Apoptosis. J. Oral Maxillofac. Surg. 20, 245–248.

[B68] Yu J. Liu Q. Lu X. Li X. Li N. Liu B. (2018). Inhibitory and Inductive Effects of Corydalis Saxicola Bunting Total Alkaloids (CSBTA) on Cytochrome P450s in Rats. Phytother Res. 32, 1818–1827. 10.1002/ptr.6117 29806105

[B69] Zeng F. L. Xiang Y. F. Liang Z. R. Wang X. Huang D. E. Zhu S. N. (2013). Anti-hepatitis B Virus Effects of Dehydrocheilanthifoline from Corydalis Saxicola. Am. J. Chin. Med. 41, 119–130. 10.1142/s0192415x13500092 23336511

[B70] Zhang C. Yao X. D. Lei F. H. (2020). Research Progress on Alkaloids in Corydalis Saxicola Bunting. Technology Dev. Chem. Industry 49, 9–13+18.

[B71] Zhou Y. Y. Chen Y. X. Zhu B. Xu X. D. (1989). Study on the Application of HPLC-Diode Array Detection in Protobeberine Quaternary Alkaloids. Chin. Traditional Herbal Drugs 20, 5–8+46.

[B72] Zhu Y. Liao J. X. (2011). Effects of Yanhuanglian Total Alkaloids on Bcl-2 Activity of Oral Squamous Carinoma Cell Lines. J. Oral Maxillofac. Surg. 21, 96–98.

[B73] Zhuge M. L. Jiang W. Z. Xiao P. Huang X. Y. (2019). Experimental Study on Anti-inflammatory and Analgesic Effects of Corydalis Saxicola Rectal Suppository. Chin. J. Ethnomedicine Ethnopharmacy 28, 11–13.

